# Seurat function argument values in scRNA-seq data analysis: potential pitfalls and refinements for biological interpretation

**DOI:** 10.3389/fbinf.2025.1519468

**Published:** 2025-02-12

**Authors:** Mikhail Arbatsky, Ekaterina Vasilyeva, Veronika Sysoeva, Ekaterina Semina, Valeri Saveliev, Kseniya Rubina

**Affiliations:** ^1^ Faculty of Medicine, Lomonosov Moscow State University, Moscow, Russia; ^2^ Institute of Higher Technologies, Immanuel Kant Baltic Federal University, Kaliningrad, Russia; ^3^ Institute of Medicine and Life Science, Immanuel Kant Baltic Federal University, Kaliningrad, Russia

**Keywords:** biocentric mathematics, ScRNA-seq, dimension reduction, cell clustering, datasets integration

## Abstract

Processing biological data is a challenge of paramount importance as the amount of accumulated data has been annually increasing along with the emergence of new methods for studying biological objects. Blind application of mathematical methods in biology may lead to erroneous hypotheses and conclusions. Here we narrow our focus down to a small set of mathematical methods applied upon standard processing of scRNA-seq data: preprocessing, dimensionality reduction, integration, and clustering (using machine learning methods for clustering). Normalization and scaling are standard manipulations for the pre-processing with LogNormalize (natural-log transformation), CLR (centered log ratio transformation), and RC (relative counts) being employed as methods for data transformation. The justification for applying these methods in biology is not discussed in methodological articles. The essential aspect of dimensionality reduction is to identify the stable patterns which are deliberately removed upon mathematical data processing as being redundant, albeit containing important minor details for biological interpretation. There are no established rules for integration of datasets obtained at different sampling times or conditions. Clustering calls for reconsidering its application specifically for biological data processing. The novelty of the present study lies in an integrated approach of biology and bioinformatics to elucidate biological insights upon data processing.

## 1 Introduction

The advent of high-throughput methods has enabled the accumulation of vast amounts of biological data over the last decades. Data analysis has evolved progresively into an extremely complex, time-consuming, and labor-intensive processing. Meanwhile, mathematical methods have been repurposed for biology as a commonly accepted standard, with the responsibility for the implementation resting with the scientists who initially applied them in biology. The hallmarks of modern science, by and large, are grounded in the credibility of the available methods and tools harnessed by all members of the scientific community. Therefore, the initial application of the purely mathematical methods in biology formed an unquestioned standard.

Gradually, as the large amount of biological information has been accumulated, numerous scientists started sharing their results on the publicly accessible platforms dedicated to biological data analysis. Many of the researchers who utilize libraries and packages implementing the functionality of key mathematical methods pointed out that the results of the well-studied and well-acknowledged data analyses were not always reproducible when applying the mathematical methods solely. Despite these observations, the scientific community did not propose any changes in approaching the biological data analysis even though biological data are fundamentally different from other types of data. As of today, despite much criticism, dissatisfaction, and perplexity, the biological community continues to use the existing toolkit.

Of note, the scRNA-seq method allows for quantifying mRNA levels within cells; however, this does not necessarily imply that these mRNAs will be translated into proteins. Extensive post-transcriptional regulatory mechanisms can influence the translation process, ultimately shaping the cell’s protein profile.

The essential difference of biological data lies in the fact that, in contrast to large values, even the smallest values or a tiny shift can lead to dramatic consequences within a cell or a living organism. This unique feature emerges from the “clamp” mode of the intracellular circuitry and signal transduction. The presence of a small amount of a transcription factor or a secondary messenger can completely alter cell metabolism, yet the calculation results may indicate a lower expression level of these proteins compared to the others. This is the emerging problem we encounter that is associated with applying standard mathematical approaches to biological data analysis.

A prime example of this is the RNA-velocity method. The method was developed in 2018 and represents a mathematical model of DNA processing. Initially, the only variables included in the equation were the variables characterizing the RNA splicing and degradation. After receiving abundant comments regarding the inadequacy of the obtained results, the developers added the variables related to vesicular transport (export from the cell) to the equation. These changes significantly increased the accuracy and reproducibility of the results obtained.

The RNA-velocity method was first introduced in 2018 and represents a mathematical model that utilizes both unspliced and spliced transcripts to predict the dynamic future expression of genes at the single-cell level. This innovative approach has been widely recognized for its capability to infer the direction of cellular differentiation and temporal dynamics of cell states. The foundational paper by La Manno et al., 2018, provides a detailed explanation of the RNA-velocity concept, algorithms, and practical applications (La Manno et al., 2018).

The field of systems biology, aiming to integrate different types of biological data, is one of the stages in the evolution of the universal mathematical methods applied for biological data analysis. The multidimensionality of biological data allows for the discovery of new hidden regularities, enabling the development of effective bio-centric computational methods.

The study consists of 4 sections describing the key stages of scRNA-seq data processing: I Pre-processing; II Dimensionality reduction from a Mathematician’s Perspective; III Cell clustering; IV Data integration from a Mathematician’s Perspective. Each section provides an explanation of the mathematical transformations occurring at this stage for biologists, as well as an example of the application of the mentioned tools and a discussion of possible ways to overcome challenges encountered during scRNA-seq data analysis.

## 2 Materials and methods

Cell Ranger version 7.0 was employed for scRNA-seq data analysis. Notably, beginning with version 7.0, Cell Ranger incorporates intronic reads by default in UMI counting - a significant shift from earlier versions, which excluded intronic reads from the UMI analysis.

For preprocessing, the web_summary.html file from the Cell Ranger’s outs folder was used. The Recluster function of the Loupe Browser tool was utilized for filtering low-quality cells. As a comparison, the Seurat package for processing scRNA-seq data was employed. Dimensionality reduction was carried out using the RunPCA function. Visualization of the results of linear dimensionality reduction method (PCA) was achieved using t-distributed Stochastic Neighbor Embedding (t-SNE) and Uniform Manifold Approximation and Projection (UMAP). Cell clustering was performed using the Find Neighbors and Find Clusters functions within the R package Seurat. Data integration was executed using the Integrate Data and Find Integration Anchors functions of the R-package Seurat.

We utilized Seurat version 4.0.6 to perform the analysis of scRNA-seq data.

## 3 Results and discussion

### 3.1 Pre-processing

**FIGURE 1:**
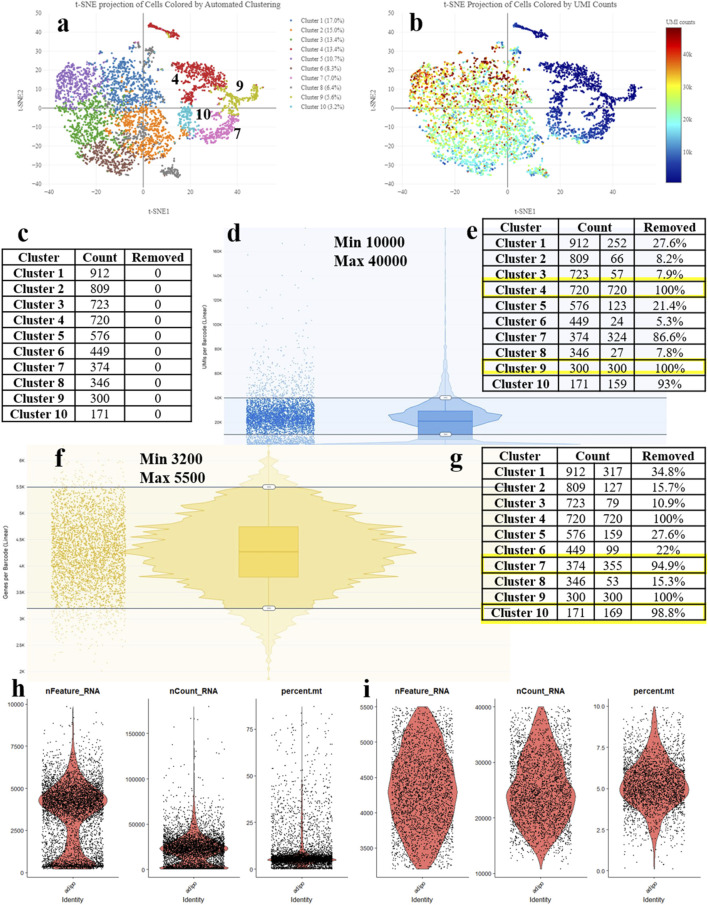
Processing the scRNA-seq data and detecting the low-quality cells during adipogenic differentiation of mesenchymal stem cells (MSCs). **(A)** t-SNE Projection of Cells Colored by UMI counts (web_summary file, CellRanger). **(B)** t-SNE Projection of Cells Colored by Automated Clustering (web_summary file, CellRanger). **(C)** Cell counts by clusters. **(D)** Removal of cells with excessively high or low counts. **(E)** Clusters removed based on UMI counts. **(F)** Removal of cells with excessively high or low features. **(G)** Clusters removed based on features. **(H)** Violin plot (VlnPlot) before cell filtering. **(I)** Violin plot (VlnPlot) of cells after cell filtering using parameters obtained from the Loupe Browser.

One of the critical data preparation steps at the preprocessing stage is cell filtering (Luecken and Theis, 2019). The outcome of the entire subsequent analysis depends on the decisions made at this stage. Currently, there are no universally accepted recommendations for the preprocessing (You et al., 2021; Ahlmann-Eltze and Huber, 2023). Most researchers make their decisions based on their own judgment (drawing from their experience of analyzing their own data). If the experiments and analysis are conducted in a laboratory either by an inexperienced researcher or for the first time, the biological interpretation of the results and the overall planning of further experiments can be significantly impaired. Depending on the implementation of this step, filtering low-quality cells can be done in various ways. In the widely used Seurat platform, three metrics are used to remove low-quality cells - the number of genes in the cell, the number of reads per cell, and the percentage of mitochondrial genes (Figures 1H,I) (Hao et al., 2024). It is considered that a low number of genes and reads in a cell, as well as a high percentage of mitochondrial genes, indicate significant cell damage. However, such indicators may imply a specific functional state of the cell, and such cells should not be removed from the analysis. The 10x Genomics platform contains a specialized software named 10x Genomics Loupe Browser, which allows for preprocessing and removal of low-quality cells based on gene count, reads, and the percentage of mitochondrial genes. Both Seurat and the 10x Genomics Loupe Browser offer valuable tools for cell filtering, each with its distinct advantages. Seurat excels in providing flexibility through adjustable parameters and iterative filtering, enabling more precise control over dimensionality reduction and clustering processes. However, it does not offer real-time feedback on cell quality, a feature uniquely provided by the Loupe Browser. The Loupe Browser’s intuitive visual interface and dynamic tracking of cluster changes significantly enhance biological relevance, making it an ideal choice for researchers prioritizing the preservation of biological context.

The approach outlined in this study integrates established tools and metrics for scRNA-seq data processing, aiming to improve practical applicability and adaptability across varied experimental contexts. Future studies will benefit from a more systematic strategy to boost reproducibility, incorporating rigorous statistical frameworks for assessing UMI and other biological indicators, while complementing visual evaluation with quantitative methods. Currently, we are developing stricter criteria for parameter selection and data analysis, aiming to align with and integrate into standards for biological interpretation.

Here, we present a generic approach for processing scRNA-seq data and detecting the low-quality cells. For this stage, we developed our original method allowing for an accurate detection of the appropriate number of cells and genes to retain.

The main principle of the methodology resides in the sequential cell filtering using the reclustering tool in the 10x Genomics Loupe Browser. Figure 1A illustrates the idea that all cell clusters can be separated into two groups. Such a segregation may be related to several reasons; however, in this case, it can be explained by the quality of cell sequencing (Figure 1B) – the cells in blue color represent problematic cells and have less than 10,000 UMIs. Low-quality and dying cells exhibit an extensive mitochondrial contamination due to a severe impact that had destroyed not only the plasma cell membrane but the two mitochondrial membranes as well. To filter them out, a filtration based on the UMI count per cell with visual control (Figure 1C) is required. By adjusting the upper and lower sliders in Loupe Browser using Recluster algorithm, we filter out cells containing more than 10,000 UMIs and less than 40,000 UMIs (Figure 1D). By moving the sliders, we change the cell counts in each cluster. Figure 1E demonstrates that by applying this procedure, we remove all cells from clusters 4 and 9 indicating that these cells were of low-quality.

Before the UMI filtration, 5,380 cells have been detected. After filtering out poorly sequenced cells, 3,328 cells remain, with 2,052 being removed (38% removed barcodes). Clusters 4 and 9 are excluded from further analysis (Figure 1E). The number of unique molecular identifiers (UMIs) per barcode can vary and it depends on the sequencing conditions. Barcodes with a very high UMI count may represent multiple cells combined together (multiplets), while barcodes with very low UMI count may represent the low-quality or empty cells (empty droplets).

The next stage represents the cell filtering based on the number of genes expressed (Figure 1F). Adjusting the sliders allows for clusters (7 and 10) with a low number of genes to emerge. Figure 1H presents violin plots of the same sample before filtering, showing a significant number of cells standing out from the main cluster. Figure 1I displays the violin plots after filtering, demonstrating an even cell distribution.

After cell filtering based on gene expression, 3,002 cells remain for further analysis with 328 cells being removed. Therefore, clusters 7 and 10 are totally excluded from further analysis (Figure 1G). Low-quality cells and empty droplets typically have a very low number of genes. Cell doublets or multiplets, on the other hand, have an excessively high number of genes.

That being said, the proposed filtering approach results in 3,002 cells remaining in the analysis out of the initial 5,380 cells, followed by 44% cell removal. The question of whether it is a high or a low proportion still remains. Considering the cost of the reagents for library preparation and subsequent sequencing, the researchers strive to retain as many cells as possible for further analysis. However, cells added by adjusting the minimum and maximum thresholds often prove to be useless in subsequent analysis as they do not fit into the biological interpretation, falling outside the main cell characteristics of the sample. To perform a comprehensive analysis of doublets in the data, it is essential to consider all currently available approaches. These include filtering cell barcodes by UMI counts, filtering cells by the number of features, filtering cells by the proportion of mitochondrial (mt) reads, filtering cells by detecting the doublets using community tools, identifying and removing empty droplets based on expression profiles, and eliminating ambient RNAs associated with barcodes.

The approach presented in this study offers a novel systematic framework for cell filtering, significantly enhancing the reproducibility and accuracy of data analysis. By integrating rigorous statistical tools to assess UMI and biological indicators complementing visual evaluation with quantitative methods. The results achieved through this methodology exhibit higher accuracy in identifying and excluding low-quality cells compared to conventional filtering techniques, such as Seurat, thereby ensuring greater biological relevance and robust substantiation of conclusions.

For visualization and controlled filtration, the Recluster function of the Loupe Browser tool represents a convenient tool. Unlike filtration in the Seurat package, the Loupe Browser allows real-time tracking of the number of cells in clusters, enabling the retention of the most biologically significant clusters. Thus, in the Seurat package, it is not possible to assess the impact of the filtration on the number and size of clusters, which may lead to an unreasonably large number of clusters and their meaninglessly small size, therefore complicating subsequent stages of scRNA-seq data analysis such as cell typing, trajectory inference, and RNA-velocity.

Herein, we primarily focus on integrating biological and bioinformatic approaches for data processing to achieve a more comprehensive understanding of biological processes. Leveraging the advanced functionalities of the Seurat package, including iterative filtering and rerunning dimensionality reduction and clustering, will further enhance the reproducibility and objectivity of the analyses. Incorporating systematic criteria based on Seurat’s infrastructure into the proposed methodology will facilitate the development of more quantitatively grounded and reproducible guidelines. Although the presented methodology already establishes a robust framework for advancements, incorporating Seurat’s capabilities to calculate quality control metrics for clusters further enhances both accuracy and objectivity.

To infer, it is essential to recognize the profound impact that preprocessing steps can have on the biological interpretation of data. Variability in interpretation can stem from how low-quality cells are filtered and analyzed. Wang et al. (2020) demonstrated that different preprocessing methods can yield significantly varied effects on clustering algorithms. Similarly, You et al. (2021) highlighted that while several packaged preprocessing workflows offer users convenient tools, their comparative performance and influence on downstream analysis remain underexplored. Additionally, Hsu and Culhane (2020) provided a comprehensive guide to PCA and related methods. In the current study, we addressed the relationship between PCA and singular value decomposition, the differences between correlation and covariance matrices, the effects of scaling, log-transformation, and standardization, as well as how to recognizу artifacts like the horseshoe or arch effect in PCA.

### 3.2 Dimensionality reduction from a Mathematician’s perspective

Dimensionality reduction methods emerged in the early 20th century and have continued to evolve, often independently in multiple fields, ultimately giving rise to a myriad of overlapping terminology (Meng et al., 2016).

#### 3.2.1 Linear dimensionality reduction method

Principal Components Analysis (PCA) is an exploratory multivariate statistical technique for simplifying complex data sets. Principal components analysis has been used to solve a wide range of biomedical problems, including microarray data analysis in search for outlier genes as well as the analysis of other types of gene expression data (Raychaudhuri et al., 2000).

scRNA sequencing yields a large number of gene patterns related to the investigated cells. Each gene pattern is defined by high dimensionality. To further analyze the data, it is necessary to group cells with similar gene patterns into the defined and separated clusters. The most common approach to scrutinizing high-dimensional data spaces is to reduce the dimensionality. Since the surrounding world is three-dimensional, it is necessary to reduce the number of variables in the dataset to three, capturing only the main characteristics of the cells. In an attempt to interpret the enormous amount of gene expression data generated by scRNA-sequencing procedures, we have developed an analytical framework by employing the statistical concepts of PCA (Craig et al., 1997). To accomplish this, PCA, which transforms the original variables into new axes, has been enrolled since the primary aim of using principal components is dimensionality reduction.

PCA is one of the most commonly used dimensionality reduction techniques in biology. It was shown that the first few PCs are closely related to the tissue of origin and that projection onto the first two PCs provides us with an informative way of visualizing this extremely high-dimensional data. Since the PCs are ordered by the amount of variance they explain—the first PC explains most of the variance, the second PC explains the second most of the variance, and so on—the researchers often choose the first few PCs, dismissing information that might be hidden in the other PCs (Huang et al., 2022). Each of these PCs may stand for biological processes, which involve various biological functions and rely on the activation or inhibition of a subset of genes. Several studies have already shown the value of Independent Component Analysis (ICA) in the gene expression context, notably Liebermeister, who was the first to apply ICA to gene expression data (Gorban et al., 2008). Comparing to PCA, ICA is considered to be a more effective method of eliminating a wide range of noise.

When selecting the number of principal components using data-driven statistics, we emphasize the importance of employing robust techniques to optimize the analysis of scRNA-seq data. While we primarily utilized the Seurat package, which offers the dims argument in the FindNeighbors function to specify the number of components, this approach does not inherently determine the optimal number of components.

To address this, incorporating methods such as the Elbow plot, which evaluates the variance explained by each component, and cross-validation techniques to assess the robustness of clustering results across varying numbers of principal components, can ensure that the selection is both statistically justified and biologically meaningful.

Of note, neither PCA nor ICA can overcome both the high dimensionality and noisy characteristics of biological data. On the other hand, PCA can occasionally fail to accurately reflect our knowledge of biology for the following reasons: a) PCA assumes that gene expression follows a multivariate normal distribution, while the recent studies have demonstrated that microarray gene expression measurements instead follow a super-Gaussian distribution, b) PCA decomposes the data based on the maximization of its variance. In some cases, the biological question may not be related to the highest variance in the data (Yao et al., 2012).

While scRNA-seq and microarrays are fundamentally distinct platforms with unique technical features and inherent biases, comparing them offers valuable historical insight into the evolution of dimensionality reduction techniques such as PCA, which were widely applied in microarray analysis. However, drawing direct parallels between these two platforms remains inappropriate given their distinct methodologies and applications.

This idea can be demonstrated with a trivial example. Supposingly, a hypothetical study comprises only three genes for analysis and cell description. For each cell, we have a set of three numbers representing the gene counts in that cell. These number sets can be represented as points in a three-dimensional space. The original information will be redistributed onto three components - in line with the dimensionality of the task. Essentially, this means that instead of using the original cartesian coordinate system, we choose a different coordinate system for our three-dimensional plot, aligning the axes along the directions of the highest point variance.

Let’s suppose we want to reduce the dimensionality to two dimensions. In this case, we should discard the component that contains the least amount of information. In other words, we exclude the direction by choosing a two-dimensional projection, along which the dispersion of points is minimal as compared to the remaining two.

In fact, a projection plane can be considered as a two-dimensional « screen » positioned to provide the minimal “image” distortion of information. By selecting the image with the maximum dispersion of the points on the plane among all possible projections, we lose less information as compared to formally dropping the last coordinate.

In general, if we aim to obtain a two-dimensional image for an n-dimensional task, we first use mathematical statistical methods to determine n directions corresponding to the maximum variance of the points. Next, we sort the directions by variance (from high to low) and discard the last n-2 directions. The resulting image does not contain the original information about the cell composition but only reflects the degree of “similarity” between the original gene sets. Actually, the larger the n value, the more information is lost during the dimensionality reduction. Therefore, the obtained image can only be used to formulate a hypothesis that needs to be further verified using other methods.

That being mentioned before, we can interpret the gene sets for each cell as points in a n-dimensional space. To perform clustering, the reduction of the dimensionality to two or three is needed to enable visualization.

#### 3.2.2 Non-linear dimensionality reduction and visualization method

PCA is called a linear dimensionality reduction method, as new coordinates are determined as linear combinations of the old ones. In other words, each new coordinate is calculated as the sum of the old ones multiplied by certain coefficients. One of the fundamental differences between non-linear methods such as t-distributed Stochastic Neighbor Embedding (t-SNE) and Uniform Manifold Approximation and Projection (UMAP) as contrasted with PCA is that they aim to preserve the similarity (and dissimilarity) between cells in a low-dimensional space. PCA, on the contrary, aims to preserve the global data structure. Let’s explain the principles of their operation by considering a two-dimensional plane as the low-dimensional space, keeping in mind that the argumentation stays the same for a three-dimensional space.

#### 3.2.3 Stochastic Neighbor Embedding (t-SNE)

The implementation of t-SNE consists of two principal stages. First, t-SNE establishes pairwise associations between all points in the n-dimensional space, assigning each point with index i a number pij, which represents the probability that this point is similar to the point with index j. The probability is calculated using a formula that incorporates the coordinate values of points i and j. As a result, similar points will have a higher probability of being selected, while the probability of selecting dissimilar points will be low. The outcome is an undirected graph with n vertices and edge lengths pij.

Next, t-SNE constructs a similar probability distribution qij for the points on the plane. In other words, first n points are defined at random on the plane (number of points is the same as the number of the investigated cells, however at this stage each point has only two coordinates instead of n). The algorithm shifts these points on the plane, aiming to position them in such a way that the probabilities of similarity of points on plane qij would be as close as possible to the corresponding probabilities pij. Actually, the method simulates each cell as a two-dimensional point, with similar cells being modeled as closely located points while dissimilar cells modeled as points that are located far apart. Thus, the method preserves the distances between points in the original n-dimensional space. The main criteria of the optimal points distribution in 2D space is a minimum of some quantity defined as a “sum of differences” of pij from qij for all i and j, and recalculated after any shift of the points on the plane.

Laurens van der Maaten and Geoffrey Hinton (Maaten and Hinton, 2008), who created this method, proposed the following physical analogy for the algorithm operation: all points are connected by springs. The stiffness of the spring connecting points i and j depends on the difference between the similarity of the two points in the multidimensional space and the two points on the plane. If the system is “released,” after a while, it will reach an equilibrium, which represents the desired distribution. The resultant force will contract the points of the two-dimensional space for the nearby points in the multidimensional space and repel them for distant points.

#### 3.2.4 Uniform manifold approximation and projection (UMAP)

UMAP was created by Leland McInnes and co-authors (Healy and McInnes, 2024) as an alternative to t-SNE and combines the advantages of both methods: t-SNE in terms of dimensionality reduction and PCA - in terms of speed. Another advantage of UMAP is that it aims to preserve not only local but also global distances between points. This shows that UMAP is not limited to the dimensionality of the original space, while t-SNE is subject to the so-called “curse of dimensionality.” The latter refers to the exponential increase in the number of combinations when trying all possible coordinates of points on the plane as the dimensionality (n) grows. As a result, t-SNE may suffer from performance issues. The benefit of UMAP is that, unlike t-SNE, which considers all possible pairwise comparisons, it only compares each point with its (k) nearest neighbors. When comparing PCA, t-SNE, and UMAP, it can be stated that tasks that are well handled by PCA are equally well handled by t-SNE and UMAP. However, the reverse is not generally true.

PCA is widely utilized for initial exploratory analyses due to its time efficiency and ability to capture global variance structures, making it an optimal choice for datasets where identifying broad variance patterns is crucially important. However, PCA may fall short in datasets with complex, non-linear relationships, as it can obscure biologically meaningful clusters. In contrast, methods such as t-SNE and UMAP excel at capturing local structures, often unveiling finer substructures or rare cell populations within a dataset. t-SNE and UMAP are particularly valuable when the primary objective is to delineate distinct cell clusters, such as in immune cell subtype analyses, where subtle differences between populations might be overlooked with PCA. For instance, t-SNE has proven effective in distinguishing closely related immune cell subpopulations that PCA might otherwise conflate. Additionally, UMAP often surpasses t-SNE in large datasets due to its faster computation and its ability to retain some global structures, making it a powerful tool for lineage tracing in developmental biology research.

These practical considerations highlight the importance of selecting the appropriate dimensionality reduction technique based on the specific characteristics and analytical objectives of the dataset at hand.

#### 3.2.5 Spatial arrangement of clusters

When specifying the dimensionality in the arguments of the RunTSNE and RunUMAP functions (Table 1), additional coordinates in the third dimension can be obtained providing a clear understanding of the spatial arrangement of clusters in relation to each other.

**TABLE 1:** Used Seurat function.

Function	Arguments
Linear dimensionality reduction	
RunPCA	npcs
Non-linear dimensionality reduction	
RunTSNE	dims
RunUMAP	dims

**FIGURE 2:**
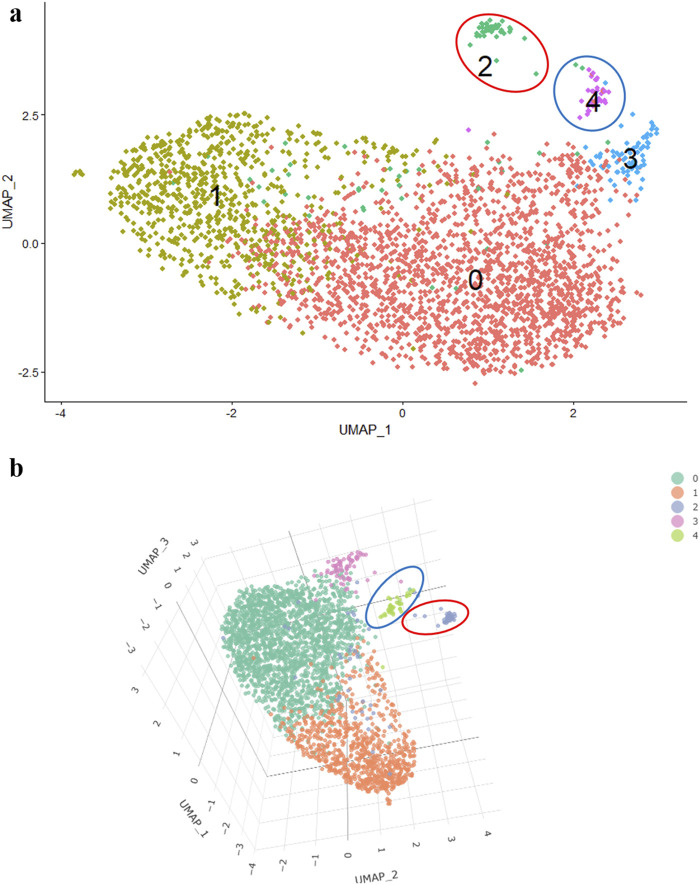
2D- UMAP **(A)** and 3D- UMAP **(B)** of MSCs induced towards adipogenic differentiation.

For example, in a 2D image, it is not evident whether the third cluster belongs to the main cluster of cells or not (Figure 2A). However, in a 3D visualization, it becomes apparent that the 3rd cluster is a part of the main cluster, while the 2nd and 4th clusters are located at a significant distance (Figure 2B).

Of note, trellis plots may offer a more effective alternative for visualizing variable pairs in a 2D space, providing clearer and more accessible data interpretation while minimizing distortions associated with 3D representations. Adopting such approaches can significantly enhance result interpretation and ensure a more accurate representation of the findings.

#### 3.2.6 Problems and limitations of dimensionality reduction

Despite a widespread use of dimensionality reduction algorithms, such as t-SNE and UMAP, these algorithms have characteristics that inherently lead to the lack of trust: they do not preserve the important aspects of high-dimensional structure and are sensitive to arbitrary choices of a user.

Though such approaches have proven valuable, they do have limitations. In particular, it is left up to users to unpack the biological meaning of the results on their own. Moreover, these methods can lead users to ignore important information hidden in higher dimensions (Table 2). The question remains how one can decide which of the reduced dimensions are biologically relevant and which can be traced to artifacts or noise (Simmons et al., 2015).

**TABLE 2:** Problems and limitations of dimensionality reduction.

Problems and limitations	Ref.
High dimensionality and noise characteristics of biological data	Raychaudhuri et al. (2000)
PCA assumes that gene expression follows a multivariate normal distribution	
PCA decomposes the data based on the maximization of its variance	
Do not preserve important aspects of high-dimensional structure and are sensitive to arbitrary user choices	Hao et al. (2024)
Researchers often choose the first few PCs, completely ignoring information that might be hidden in other PCs	
It is left up to the user to unravel the biological meaning of the results on their own	Huang et al. (2022)
These methods can lead to users ignoring important information hidden in higher dimensions	
In lacking an interpretable link between the data’s features and low-dimensional representation, their use as hypothesis-generating tools is restricted	Yao et al. (2012)
Application of only one method does not suffice to capture all important signals	Gorban et al. (2008)
Biological world seems much more complex than the world of celestial mechanics	Eckmann and Tlusty (2021)

Moreover, it is worth mentioning the importance of additional parameters in methods such as RunPCA, RunTSNE, and RunUMAP. For instance, the “features” parameter in RunPCA, which dictates which genes are considered during principal component calculations, plays a critical role in shaping the analysis. Similarly, the ability to select the input space in both RunTSNE and RunUMAP has a substantial influence on the resulting embeddings and, consequently, on downstream analyses.

Of note, the application of only one method often does not suffice to capture all important signals. It is therefore necessary to apply several methods addressing different aspects of the data under scrutiny. Lehrmann, A et al., developed a framework for linear and non-linear dimension reduction methods within our visual analytics pipeline SpRay (Lehrmann et al., 2013). Since an interpretable link between the data’s features and low-dimensional representation is missing, we cannot rely on them as hypothesis-generating tools (Boileau et al., 2020).

In other words, it is essential to understand that mathematical methods applied for biological data processing and visualization are used for data analysis in other scientific disciplines. Insufficient insight into the mathematical algorithm behind each stage of raw data transformation forces the biologists to regard the obtained results as given, without considering alternative representations. Meanwhile, when the parameters are altered, the patterns identified during the initial analysis are preserved, albeit with slight variations. The preservation of these patterns indicates the presence of specific interrelations among the elements of the dataset. The skill of interpreting the obtained results is gradually developed through professional communications with colleagues regarding the identified patterns. However, in most cases, the obtained results confirm or reflect the initial expectations, but the most valuable findings could be those that contradict the existing notions, sometimes even overturning them. In any case, every result obtained through bioinformatic methods needs to be subsequently validated experimentally.

Dimensionality reduction is indispensable for scRNA-seq data processing; however, its blind application can lead to incorrect interpretation of the obtained results. Having analyzed a substantial amount of our own data, we arrived to a conclusion that for a more accurate biological interpretation of the obtained results at this stage, it is essential: to use coordinates of other components besides 1 and 2; to utilize three-dimensional data representation to avoid incorrect cluster arrangement relative and employ different parameter values in the functions to search for the variant of results that best describe the subject of study.

To ensure clarity and avoid any confusion, the terms can be precisely defined as follows:

• PCA Components: Linear combinations of the original variables designed to capture the maximum variance in the dataset. In scRNA-seq analysis, PCA components are frequently employed to reduce dimensionality while retaining key patterns in gene expression.
• UMAP Dimensions: UMAP (Uniform Manifold Approximation and Projection) projects high-dimensional data into a lower-dimensional space, preserving both local and global structures. Dimensions in UMAP refer to the axes of this reduced space, which are used for visualization and downstream analysis.
• t-SNE Dimensions: t-SNE (t-distributed Stochastic Neighbor Embedding) is a non-linear visualization method focused on preserving local similarities between data points in a lower-dimensional space. t-SNE dimensions correspond to the axes in this reduced visual representation.

### 3.3 Cell clustering

Clustering is another crucial step in biological data processing. It is different from classification since the elements under investigation are being grouped based on a set of certain features within clustering process. Biological analysis considers cell samples as elements under examination, while the expressed genes are referred as certain features (Duò et al., 2018).

For novice researchers, the varying number of clusters obtained during data processing and analysis can be quite challenging (Yu et al., 2022; Zhang et al., 2023). At a first glance it may seem that the more clusters one can obtain, the more information can be gleaned about the presence of different cell types in a sample, or cells at different stages of differentiation, or cells with specific biochemical processes being activated at the time of sequencing. Still, this view is highly questionable as discussed below.

The R package Seurat allows for the modification of argument values of functions for finding the nearest neighbors and clustering (Table 3). In the FindNeighbors function, the dims argument allows for changing the number of principal components for analysis, while the FindClusters function has the option to select different clustering algorithms. The FindNeighbors and FindClusters functions from the Seurat package offer exceptional flexibility for handling scRNA-seq data. These functions effectively address challenges posed by the unevenness and diversity of scRNA-seq datasets through featuring the options to select clustering algorithms and adjust the number of principal components. They are indispensable tools for managing complex data, enabling detailed investigations and uncovering essential biological signals within cell populations. Unlike methods such as hierarchical clustering or DBSCAN, Seurat’s functions are specifically designed to accommodate the unique characteristics of scRNA-seq data, allowing for more effective management of their sparse and high-dimensional nature.

**TABLE 3:** Used Seurat function.

Function	Arguments
Find Neighbors (Waltman and van Eck, 2013)	Dims
Find Clusters (Traag et al., 2019)	Resolution
	Original Louvain algorithm
	Louvain algorithm with multilevel refinement
	SLM algorithm
	Leiden algorithm

**FIGURE 3:**
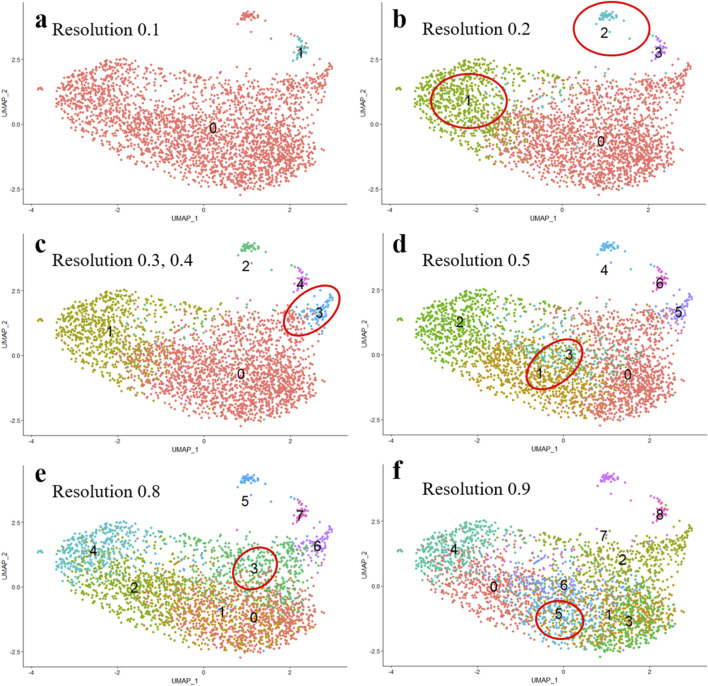
Resolution-dependent variations in the number of clusters identified in mesenchymal stem cells (MSCs) undergoing adipogenic differentiation. **(A)** Resolution 0.1, **(B)** Resolution 0.2, **(C)** Resolution 0.3, 0.4, **(D)** Resolution 0.5, **(E)** Resolution 0.8, **(F)** Resolution 0.9.

To determine whether increasing the number of clusters is reasonable, several monitoring variations need to be applied and tested. First, as demonstrated in Figure 3 the number of clusters grows (from 2 to 9) along with the increase in resolution (Figures 3A–F). However, even the initial analysis of cluster number apparently indicates that with the increase in resolution, clusters 1 and 2 emerge within cluster 0 (Figure 3B), while cluster 3 appears to be distinguishable in addition to clusters 1 and 2 (Figure 3C), clusters 1 and 3 become evident at the center of the residual cluster 0, (Figure 3D) and etc. Upon increasing the resolution, some clusters remain indistinguishable within the next several steps, while others appear to be resolved as early as the second step. The observed variations are biologically significant, as they allow us to identify clusters that are resistant to clustering and are likely to contain specific features, enabling the identification of cells of a particular type, or differentiation stage, or reflecting distinct biochemical processes.

Second, the number of cells within a cluster makes a difference. As demonstrated in Table 4, in case the number of clusters is equal to 2, all cells are distributed into two groups - 2893 (cluster 0) and 34 (cluster 1) cells. If the number of clusters is equal to 4, cluster 0, which initially contained 2893 cells, is now divided into three clusters with cell counts of 200, 806, and 77. Of note, the 4th cluster retains 35 cells, which is 1 cell less than when the cell sample is divided into two clusters. To infer, the biologically significant cells are mainly located in clusters that maintain their size upon the increase of the resolution parameters.

**TABLE 4:** Cell number in at different resolution parameters.

Resolution	Number of clusters	Number of cells in clusters								
		0	1	2	3	4	5	6	7	8
0.1	2	893	34	—	—	—	—	—	—	—
0.2	4	2009	806	77	35	—	—	—	—	—
0.3	5	935	805	79	73	35	—	—	—	—
0.4										
0.5	7	1066	875	589	207	83	72	35	—	—
0.6										
0.7										
0.8	8	686	598	561	500	384	82	81	35	—
0.9	9	540	536	477	401	86	72	193	87	35
1										

Third, the lists of the most represented genes in each cluster at different resolutions are important. The term “most represented genes” refers to highly variable genes predominantly expressed in specific clusters, serving as potential cluster-specific markers. A principal question that remains to be answered is whether the appearance of new clusters with increasing resolution has any biological significance or whether these new clusters are related to specifying the desired number of clusters. Table 5 lists the top 10 most represented genes in each cluster at different resolutions. Clusters with similar (but not identical) sets of genes are highlighted with the same color. Visual analysis of Table 5 uncovers that some clusters are inherited from the beginning of the clustering process, while the others appear only at a certain resolution. At some point, there is an artificial splitting effect of a biologically significant cell cluster into two less meaningful ones. Significance in this case refers to the connection of all cluster genes to a specific process, while a decrease in significance corresponds to the emergence of these genes in two neighboring clusters.

**TABLE 5:** The list of the first 10 genes in each cluster at different resolution parameters.

Resolution 0.1								
DCN	CD36							
PTX3	FABP4							
SFRP2	CRYAB							
IGF1	POSTN							
MMP2	TIMP3							
LUM	IGFBP5							
COMP	COL4A1							
PDGFRA	COL4A2							
PLPP3	NDUFA4L2							
EFEMP1	MYLK							
Resolution 0.2								
LUM	ACTA2	LRRC75A	CD36					
AKR1C1	TAGLN	SCD	FABP4					
IGF1	MYL9	TGFBI	POSTN					
PLPP3	TPM1	COL4A1	CRYAB					
TWIST1	CALD1	IGFBP5	TIMP3					
RAB31	PALLD	FN1	IGFBP5					
GPX3	RGCC	LOX	COL4A1					
SAT1	MYLK	POSTN	COL4A2					
GJA1	MFAP5	FADS1	NDUFA4L2					
SRPX	ACTB	THBS1	MYLK					
Resolution 0.3–0.4								
LUM	ACTA2	LRRC75A	FABP4	CD36				
AKR1C1	TAGLN	TGFBI	FABP5	FABP4				
IGF1	MYL9	COL4A1	G0S2	POSTN				
PLPP3	TPM1	SCD	LPL	CRYAB				
TWIST1	CALD1	IGFBP5	IGFBP5	TIMP3				
MMP3	PALLD	FN1	APOE	IGFBP5				
RAB31	MYLK	LOX	PNPLA2	COL4A1				
SAT1	RGCC	FADS1	CEBPA	COL4A2				
GJA1	MFAP5	POSTN	ACACB	NDUFA4L2				
DCN	ACTB	THBS1	CRYAB	MYLK				
Resolution 0.5–0.7								
IGF1	DKK3	ACTA2	CHRDL1	LRRC75A	FABP4	CD36		
MMP3	PTX3	TAGLN	PLPP3	COL4A1	FABP5	FABP4		
PRRX1	IL1RL1	MYL9	GPX3	SCD	G0S2	POSTN		
TWIST1	MT1E	TPM1	LUM	TGFBI	LPL	CRYAB		
COL6A3	MT1X	CALD1	CFD	IGFBP5	IGFBP5	TIMP3		
OLFML2B	MT1M	MYLK	AKR1C1	FN1	APOE	IGFBP5		
SELENOP	TSC22D3	PALLD	APCDD1	LOX	PNPLA2	COL4A1		
ZFP36L2	COMP	ACTB	DCN	FADS1	CEBPA	COL4A2		
HMCN1	MORF4L2	CAV1	SPON2	COL1A1	ACACB	NDUFA4L2		
SFRP2	ITGBL1	TPM2	MT2A	THBS1	CRYAB	MYLK		
Resolution 0.8								
MMP3	AKR1C1	PTX3	PLIN2	ACTA2	LRRC75A	FABP4	CD36	
SFRP2	PLPP3	DKK3	C7	TAGLN	SCD	FABP5	FABP4	
COL6A3	CHRDL1	IL1RL1	APOE	MYL9	TGFBI	G0S2	POSTN	
PRRX3	LUM	RGCC	CLDN11	TPM1	FN1	LPL	CRYAB	
HMCN1	IGF1	MFAP5	RSPO3	MYLK	COL4A1	IGFBP5	TIMP3	
AL139393.2	MTR	COL1A1	MMP14	CALD1	FADS1	APOE	IGFBP5	
ASS1	GPX3	COMP	SCD	PALLD	LOX	PNPLA2	COL4A1	
SCRG1	DCN	MT1E	ADAM12	CAV1	IGFBP5	CEBPA	COL4A2	
COL3A1	RAB31	ELN	ADH1B	ACTB	COL1A1	ACACB	NDUFA4L2	
CXCL3	MGP	SCG2	CRYAB	CNN1	THBS1	CRYAB	MYLK	
Resolution 0.9								
PTX3	COL6A3	FABP4	IGF1	ACTA2	DKK1	CHRDL1	LRRC75A	CD36
DKK3	SFRP2	FABP5	MMP3	TAGLN	IL1RL1	PLPP3	SCD	FABP4
RGCC	ASS1	G0S2	GALNT15	MYL9	MORF4L2	GPX3	COL4A1	POSTN
IL1RL1	SCRG1	IGFBP5	LUM	TPM1	AKR1C1	CFD	TGFBI	CRYAB
COL1A1	MT1E	APOE	TWIST1	MYLK	MTR	LUM	FADS1	TIMP3
MFAP5	PRRX1	LPL	MGP	CALD1	RASSF4	AKR1C1	FN1	IGFBP5
MT1E	COL3A1	CRYAB	RAB31	PALLD	RAB31	SPON2	LOX	COL4A1
COMP	MT1X	SCD	TNFSF10	CAV1	SULF2	APCDD1	IGFBP5	COL4A2
SCG2	HMCN1	C7	FBXO32	ACTB	TSC22D3	SAA1	COL1A1	NDUFA4L2
ELN	MT2A	PLIN2	GJA1	CNN1	BEX3	DCN	THBS1	MYLK

In clustering, unlike classification, the analyzed features of elements are grouped together based on the similarity of multiple characteristics. Within the context of biology, this is of great importance because the appropriately selected parameters of the used functions and a biocentric perception of the obtained results allow for discovering previously unknown patterns. Specifically in biology, a cluster corresponds to subpopulations of cells that have similar biological process, consistently express a membrane marker, or have a certain number of transcripts reads. Therefore, in addition to automatically or manually typing the obtained clusters, we recommend generating a series of projections with different cluster numbers, evaluating the number of cells within them as well as differential expression of genes. This will help to eliminate erroneous hypotheses that may arise while interpreting the results.

To ensure consistency in clustering outcomes across various datasets, a dedicated focus on both parameter selection and data preprocessing stages is requires. A key recommendation can be to meticulously document the complete parameter settings and preprocessing workflows in a detailed and transparent manner, enabling replication in future studies. This includes specifying the resolution settings in clustering, the principal components utilized in dimensionality reduction, and the criteria for identifying and filtering low-quality cells. Moreover, to mitigate variability in clustering outcomes when analyzing new datasets, we recommend leveraging tools like Seurat—not only for their robust clustering capabilities but also for their ability to integrate datasets by aligning them basing on common features. This approach minimizes inter-dataset variability, allowing for more consistent clustering across different experiments. Additionally, sensitivity analyses on parameter choices can provide valuable insights into the critical settings required to maintain reproducibility.

Towards this end, in clustering, unlike classification, the analyzed features of elements are grouped together based on the similarity of multiple characteristics. Within the context of biology, this is of great importance because the appropriately selected parameters of the used functions and a biocentric perception of the obtained results allow for discovering previously unknown patterns. Specifically in biology, a cluster corresponds to subpopulations of cells that have similar biological process, consistently express a membrane marker, or have a certain number of transcripts reads. Therefore, in addition to automatically or manually typing the obtained clusters, we recommend generating a series of projections with different cluster numbers, evaluating the number of cells within them as well as differential expression of genes. This will help to eliminate erroneous hypotheses that may arise while interpreting the results.

### 3.4 Data integration from a Mathematician’s perspective

Let’s consider two data sets, each representing a set of vectors that reflect the expression patterns of cells in two samples. Without loss of generality, let us consider integration as identifying groups of cells within the control and experimental samples whose expression patterns are so similar that they can be defined as cells of the same type.

Data integration is preceded by several steps of data processing and analysis. First, regardless of whether integration is being performed, data normalization is conducted to structure the data with different value ranges into a unified pattern which allows for their comparison. Essentially, the process involves first shifting and then scaling the original set of values. After normalization, the expression values of all genes lie within the same range. The outliers, which are cells that are likely to be “junk” cells with some probability, are removed from the data sets and excluded from the analysis. Once the data is properly normalized, all genes should be considered equal in their potential influence, meaning none of them can be given preference or deemed more significant than the others in advance.

Next, genes are selected to assess the degree of similarity between the cells. If the number of genes is not excessively large, all genes can be considered. In Seurat, the default value for the number of genes is set to 2000 (functions Find Variable Features and Select Integration Features).

Integration is performed in two steps: defining the “anchors” (Find Integration Anchors) and integrating the cells (Integrate Data). “Anchors” represent pairs of similar cells in different samples. First, dimensionality reduction is performed (specified by the “dims” parameter). In Seurat (Kujawa et al., 2022), this can be achieved using canonical correlation analysis (CCA), reciprocal PCA, or reciprocal LSI (latent semantic indexing), as chosen by the user (Table 6) (Luecken et al., 2022). Then, each cell from one sample is compared to every cell from the other sample. If the cells turn out to be similar (known as mutual nearest neighbors), the pair is labeled as an “anchor.”

**TABLE 6:** Used Seurat function.

Function	Arguments
Integrate Data	normalization.method = “SCT”
	normalization.method = “LogNormalize”
Find Integration Anchors	reduction = “rpca”
	reduction = “cca”
	reduction = “rlsi”

As dimensionality reduction comes at the expense of accuracy, false “anchors” may arise. Therefore, after comparing all cells from both samples in low dimensionality, a filtering of “anchors” is performed using the original data or full-dimensional data, and false anchors are removed. Let’s explain the mechanism of anchor filtering. Let’s suppose an anchor “links” cell Q and cell R from two original samples Query and Reference, respectively. In the Reference sample, the number of the nearest neighbors for cell Q is determined using the parameter k.filter, which represents the most similar cells. If cell R is not among them, the anchor is removed.

Each remaining anchor is assigned a weight factor that characterizes the “quality” of that anchor. To explain the evaluation mechanism. Let’s take the anchor QR and a certain number of most similar anchors, determined by the parameter k.score. First, we obtain two sets of cells in the Query and Reference samples, consisting of the ends of these anchors. Second, we find the nearest neighbors of cells Q and R, resulting in two additional sets. The more identical cells are present in these pairs of sets, the higher weight is assigned to the anchor QR. In other words, anchors should link the sets of similar cells in one sample with sets of similar cells in the other sample. Next, the integration is performed, which involves merging pairs of cells labeled as a single anchor, taking into account their weight.

### 3.5 Description and comparison of methods

**FIGURE 4:**
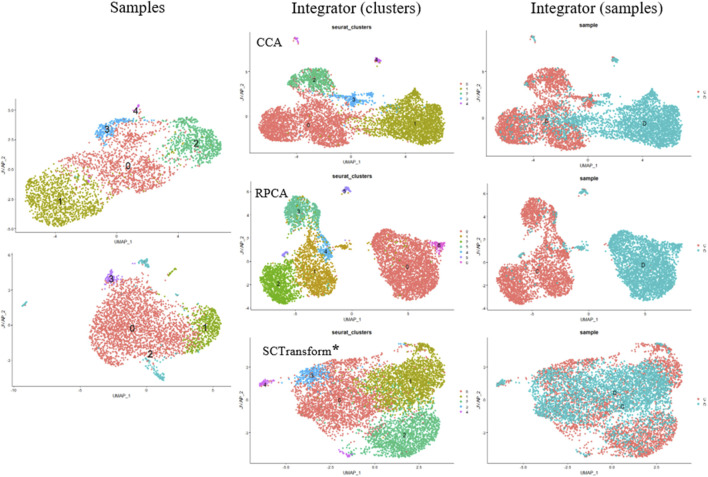
Cell Clustering and Integration Methods Using the Seurat Package. Cell Samples: Upper left–MSCs induced for adipogenic differentiation; lower left–control MSCs sample. Integrator (clusters): Examples of data integration and clustering methods include CCA (Canonical Correlation Analysis), RPCA (Reciprocal PCA), and SCTransform (Regularized Negative Binomial Regression applied to normalize UMI count data) (*SCTransform is not an integration method, it is used for data normalization as a substitute for Normalize Data, Find Variable Features, and Scale Data functions. Integrator (samples) represents the integrated object of overlapping cell samples (control cells and MSCs induced for adipogenic differentiation).

Small modifications have been made to integrate scRNA-seq datasets into the algorithm. Instead of using traditional CCA (Canonical Correlation Analysis) to determine anchors, we employ randomized non-linear PCA analysis called RPCA. When searching for anchors between any two datasets using RPCA, each dataset is projected into the space of the other principal components, and anchors are selected based on pre-established requirements of mutual neighborhood. The commands for both algorithms are quite similar, but these two methods can be applied in different situations (Figure 4).

CCA (Canonical Correlation Analysis) is well-suited for determining common sources of variation between datasets. In this case, cell types are well-known and remain constant, while significant differences in gene expression patterns are envisaged across different experimental conditions. CCA is used for integration when experimental conditions or external factors cause strong expression shifts. It is useful for integrating datasets containing different types of data (proteomics, transcriptomics, metabolomics, etc.) or datasets obtained from different species. However, CCA-based integration can also lead to excessive correction, especially when a large proportion of cells do not overlap between datasets. Thus, it is not recommended to use a default integration method as it may not fit the specific characteristics of the cells being studied.

At this stage, the interaction between bioinformaticians and biologists is crucial in selecting an appropriate method. However, it is also not advisable to rely solely on one chosen method, as even profound knowledge of the subject may not guarantee that all sequencing preparation stages are executed properly.

Of note, the RPCA-based integration runs significantly faster, and also represents a more conservative approach where cells in different biological states are less likely to “align” after integration.

Another method is called “sctransform,” and it avoids some of the pitfalls of the standard normalization workflows, including additional application of a pseudocount, and log-transformation. In particular, scTransform is primarily a normalization method. Within the framework of scRNA-seq analysis, data normalization, exemplified by scTransform, represents a crucial step prior to integrating different datasets. Such normalization methods are instrumental in mitigating technical variations, thereby ensuring the data is optimally prepared for subsequent analytical processes, including integration. For further clarification the readers may address SCTransform vignette (https://satijalab.org/seurat/articles/sctransform_vignette.html, n.d.). In a single command, and without any requirement to set user-defined parameters, sctransform performs normalization, variance stabilization, and feature selection based on a UMI-based gene expression matrix (Hafemeister and Satija, 2019).

Integration, as one of the stages in the scRNA-seq data processing pipeline, was not initially used. The need for such an algorithm arose due to the specifics of cell sample preparation, storage, and cell culturing *in vitro* (Hie et al., 2019). It is not always possible to design an experiment that would take into account all of the organizational issues related to cell isolation and culturing as well as cell preparation for scRNA-seq (Richards et al., 2021). Additionally, due to limited resources in research tasks, scientists are often compelled to employ approaches that save both time and resources (Ryu et al., 2023). As a result, cells may be isolated at different times, by different individuals, and cell culturing and storage as well as library preparation for sequencing are often performed without extensive experience. All these deviations can result in artifacts during integration. The goal of integration is to create an “integrated” data assay for downstream analysis, identify the shared cell types between datasets, obtain cell type markers that are conserved in both control and stimulated cells, and compare the datasets to identify cell-type-specific responses to stimulation. In addition, there may be a less common task of expanding the cell number in a sample or increasing the number of reads. All this allows for creating the integrators, once analyzed, can reveal missing patterns in the analysis of individual sample arrays. The choice of integration algorithm is a crucial stage since the subsequent downstream analysis results will depend on this very stage.

## 4 Conclusion

The immense advances that are being made in mapping gene expression at the resolution of single cells significantly extended and expanded our understanding of biological objects. Meanwhile, straightforward application of standard mathematical methods to biological data analysis has already led to several erroneous hypotheses. The underlying reasons behind this inconsistency is the lack of communication between mathematicians and biologists. The need for biological data analysis emerged long after the mathematical methods were created, so blindly using them “by default” in biology is completely unjustified. The solution lies in a close collaboration between these specialists, with biologists taking the lead in developing algorithms and equations that describe biological processes. Students studying in faculties where equal attention is given to biology and mathematics are to become the versatile experts who can develop professional software packages and libraries that take into account the peculiarities of biological entities.

The early algorithms for scRNA-seq analysis have undergone significant evolution. This evolution has been driven not only by adaptation and modification of the existing mathematical approaches for analyzing biological data but also by development of new algorithms that have noticeably expanded the modern standard pipeline for scRNA-seq data processing. However, there are key stages in the algorithm, greatly affecting the right decisions made for obtaining reliable results and the success in their interpreta-tion. Experience shows that novices in this type of data analysis often make similar mistakes. The goal of this article was to adapt the mathematical principles and methods used in scRNA-seq data analysis for application in biology. We also provide practical recommendations for conducting the main stages of scRNA-seq data analysis using the R package Seurat. One conceptual issue addressed in the article is, in our view, the blind adherence to the existing standard methods of scRNA-seq data analysis. Mathematical methods used in analysis are employed on par with biological experimental approaches. However, the complexity of biological data calls for developing tailor-made mathematical methods that take into account the peculiarities and complexity of biological systems.

Devising a new bio-centric analytical framework may be facilitated by the emerging fields in computational mathematics such as systems and algebraic biology, systems theory, and artificial intelligence. These fields will enable the researchers, in close collaboration with biologists, to mathematically describe the biological processes as well as to develop integral equations that consider both, significant and non-significant variables.

## Data Availability

The original contributions presented in the study are included in the article/supplementary material, further inquiries can be directed to the corresponding author.

## Abbreviations

t-SNE, t-distributed Stochastic Neighbor Embedding; UMIs, unique molecular identifiers; MSCs, mesenchymal stem cells; PCA, Principal Components Analysis; ICA, Independent Component Analysis; UMAP, Uniform Manifold Approximation and Projection; CCA, canonical correlation analysis; RPCA, reciprocal PCA.
